# Primary pulmonary lymphoma mimicking a refractory lung abscess: A case report

**DOI:** 10.3892/ol.2015.2929

**Published:** 2015-02-03

**Authors:** TAKESHI MATSUMOTO, KOJIRO OTSUKA, YUKI FUNAYAMA, YUKIHIRO IMAI, KEISUKE TOMII

**Affiliations:** 1 Department of Respiratory Medicine, Kobe City Medical Center General Hospital, Kobe, Japan; 2 Department of Hematology, Kobe City Medical Center General Hospital, Kobe, Japan; 3 Department of Pathology, Kobe City Medical Center General Hospital, Kobe, Japan

**Keywords:** primary pulmonary lymphoma, lung abscess, refractory

## Abstract

The current study presents a case of primary pulmonary lymphoma (PPL) mimicking refractory lung abscess that was diagnosed at autopsy. An 80-year-old male with clinically inapparent aspiration presented with a large cavitated mass and pleural effusion. A lung abscess and empyema was diagnosed, therefore, antibiotics were administered and the pleural effusion was drained. Various examinations, including a biopsy, yielded no specific diagnosis. The lesion was considered inoperable due to the poor general condition of the patient. Subsequently, the mass that had been diagnosed as a refractory lung abscess became enlarged and a repeat biopsy resulted in a diagnosis of diffuse large B-cell lymphoma. The patient succumbed to sudden respiratory failure, and the final diagnosis of PPL was confirmed at autopsy. PPL is a rare disease that accounts for 0.45% of all pulmonary malignant tumors and is difficult to diagnose in inoperable cases. Therefore, patients with PPL who do not undergo surgery can be misdiagnosed and consequently treated inappropriately. PPL should therefore be considered in the differential diagnosis of a refractory lung abscess.

## Introduction

Primary pulmonary lymphoma (PPL) is a rare disease that reportedly accounts for 0.45% of all pulmonary malignant tumors (1). The current definition of PPL covers low-grade B-cell PPL (the most common), high-grade B-cell PPL, and lymphomatoid granulomatosis (2). The incidence of PPL peaks in the sixth and seventh decades, and the male: female ratio is ~1:1 (3). Certain cases are diagnosed in small specimens, such as those obtained by bronchoscopic biopsy, and bronchoalveolar lavage (BAL) is occasionaly of use (4). However, PPL is usually difficult to diagnose in small specimens unless there are visible endobronchial lesions (5) and BAL alone does not contribute to the morphologic analysis, several studies have recommended performing surgery to make the diagnosis, which led to the treatment itself (1,6). Diagnostic radiological findings, namely tumor spread without the destruction of the existing lung structure, have been reported (2). However, these features are not initially apparent in certain cases. Therefore, patients who do not undergo surgery can be misdiagnosed and consequently treated inappropriately. The current study presents a case of PPL mimicking a refractory lung abscess.

## Case report

In April 2012, an 80-year-old male who had been institutionalized five years prior to admission to the Kobe City Medical Center General Hospital (Kobe, Japan) was referred due to a persistent low-grade fever. Prior to this referral, the patient had been treated as for right-sided pneumonia and pleuritis; however, a right-sided pleural effusion and low-grade fever persisted. The patient was admitted to the hospital with a diagnosis of a lung abscess and empyema. The medical history showed curative surgery had been performed for esophageal cancer 10 years previously and that the patient was currently receiving treatment for diabetes mellitus. The patient was a carrier of the hepatitis C virus and was suffering from pneumoconiosis. No history of tuberculosis pleuritis or an artificial pneumothorax procedure was evident.

**Figure 1:**
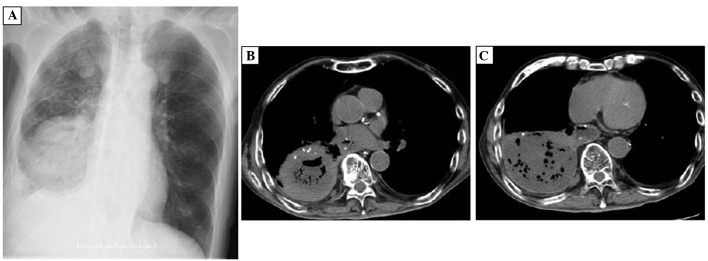
(A) Chest X-ray film on first admission showing a large mass in the right lower lung field. (B and C) Non-enhanced computed tomography scan of the chest on first admission showing a large cavitated mass in the right lower lobe and pleural effusion with multiple gas bubbles.

Upon physical examination, diminished lower right lung sounds were heard. A chest radiograph showed a large mass with multiple air-fluid levels and pleural effusion in the right lower field (Fig. 1A). Chest computed tomography (CT) showed a large mass with a cavity and fluid in the right lower lung lobe, and pleural effusion with multiple gas bubbles (Fig. 1B and C). Laboratory studies revealed a normal white blood cell count of 7,200/mm^3^, a marginal increase in neutrophils (75%; normal range, 37–72%), an increased C-reactive protein level of 3.4 mg/dl (<0.3 mg/dl) and a normal lactate dehydrogenase level of 150 U/l. Aspirated pleural fluid appeared gray-white and sludgy, with an increased lactate dehydrogenase level of 10,630 U/l (normal range, <200 U/l) and a decreased glucose concentration of 16 mg/dl (normal range, >60 mg/dl). A cell count could not be performed, as the cells had disintegrated.

With the diagnosis of a lung abscess and empyema, antibiotics (1.5 g ampicillin/sulbactam every 6 h) were administered for 21 days, and drainage and washing out of the empyema through a 20-Fr drainage tube was performed. Cultures for bacteria, including acid-fast bacteria, and the cytology of the pleural effusion were negative. Three weeks later, a CT scan showed that the lung abscess had become slightly enlarged, in spite of improvement of the pleural effusion. Bronchoscopy and gastroscopy revealed no specific lesions. Inapparent aspiration was demonstrated by videofluorography, and was considered to have caused the lung diseases and to have contributed to their exacerbation. Surgery was considered to be contraindicated by due to the poor general condition of the patient. An ultrasound-guided centesis of the lung abscess proved difficult and no biopsy sample was obtained. As the patient refused an intravenous infusion or CT-guided centesis, the antibiotic regime was changed to 400 mg oral garenoxacin (GRNX) daily. Subsequent to the patient being discharged, the lung abscess remained unchanged in size during a further two months of GRNX treatment. The patient then consented once more to CT-guided drainage of the lung abscess. Again, the cultures for bacteria, including acid-fast bacteria, and the cytology were negative. Antibiotics were discontinued for a month, during which time the lung abscess remained the same size; it was therefore believed to be scar tissue. A transfer to another hospital was completed, as the patient’s condition was no longer acute.

**Figure 2:**
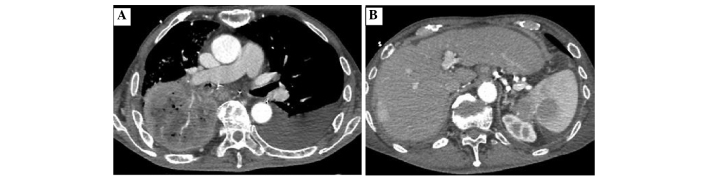
(A and B) Contrast-enhanced computed tomography scan of the chest on third admission showing that the lung mass is larger and is infiltrating the blood vessels. There is also a new solid lesion in the spleen.

**Figure 3:**
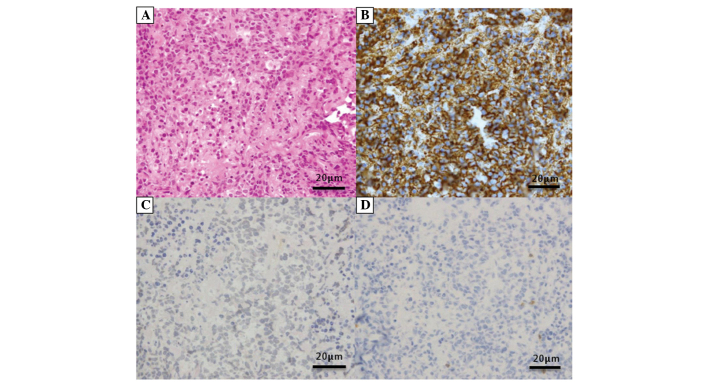
Microscopy findings on lung biopsy. (A) HE stain, showing infiltration of atypical lymphocytes. (B) Immunostaining is positive for CD20 and (C) negative for CD3 and (D) Epstein-Barr virus encoded early RNA *in situ* hybridization.

**Figure 4:**
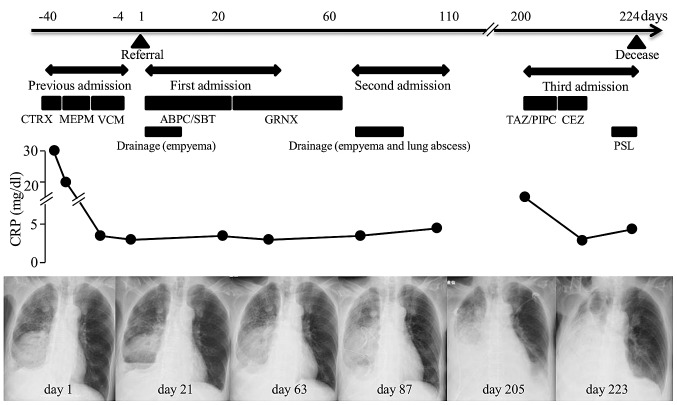
Clinical course. ABPC/SBT, ampicillin/sulbactam; CEZ, cefazolin; CTRX, ceftriaxone; GRNX, garenoxacin; MEPM, meropenem; PSL, prednisolone; TAZ/PIPC, tazobactam/piperacillin; VCM, vancomycin.

Three month later, the patient was admitted to the Kobe City Medical Center General Hospital due to respiratory failure. A CT scan showed that the lung mass had increased in size and was infiltrating the blood vessels. A new solid lesion was also identified in the spleen (Fig. 2A and B). Accordingly, lymphoma was considered in the differential diagnosis of the mass that had previously been diagnosed as a refractory lung abscess. Due to its increased size, the mass could now be biopsied with ultrasound guidance. The biopsy revealed atypical lymphocytes that were positive for cluster of differentiation (CD)20 and IgH rearrangement, and negative for CD3 and Epstein-Barr virus-encoded early RNA *in situ* hybridization, all of which resulted in a diagnosis of diffuse large B-cell lymphoma (DLBCL), stage IIIB (Fig. 3A–D). However, the general condition of the patient was too poor to tolerate aggressive treatment, and so antibiotics for bacterial pneumonia (4.5 g tazobactam/piperacillin, every 8 h for five days and 1 g cefazolin, every 8 h for nine days) and palliative steroids (20 mg prednisolone, daily for five days) were administered. The patient subsequently succumbed to sudden respiratory failure (Fig. 4).

**Figure 5:**
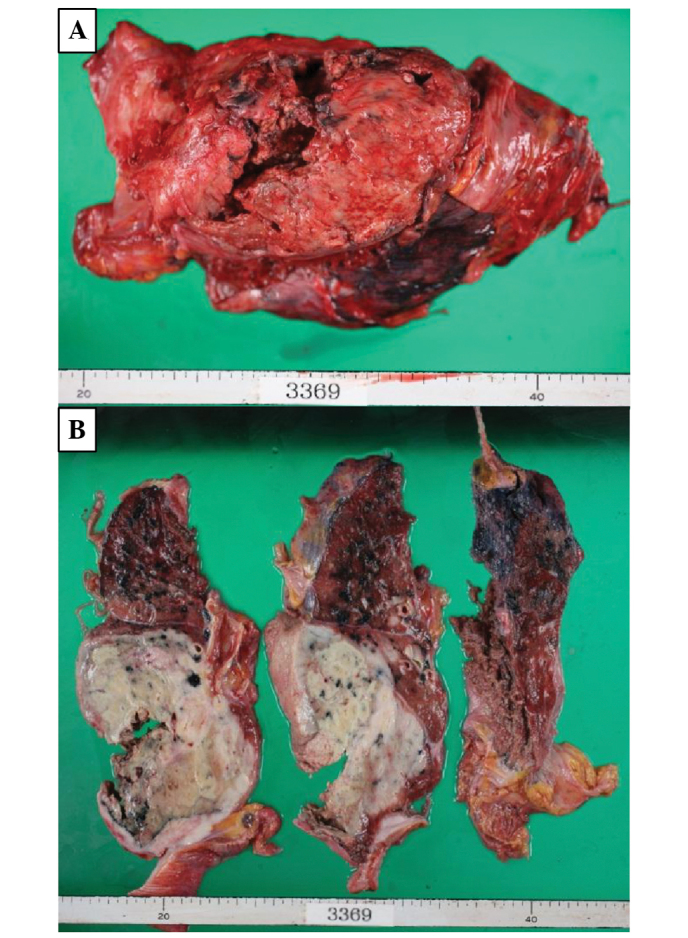
Autopsy findings. (A) The right lung showing a large cavitated mass. (B) Slices of the right lung showing an expanding large tumor displacing normal lung without infiltrating it.

An autopsy revealed multiple lymphoma lesions located in the right lung, esophagus, duodenum, ileum, mesenteru, spleen and left anterior superior iliac spine (Fig. 5A and B). As the right lung lesion was so large and the other lesions had not previously been detected, the final diagnosis was of PPL.

## Discussion

The present case of PPL, which is rare and difficult to diagnose, and was therefore misdiagnosed as a lung abscess, is reported in order to highlight the requirement for considering this diagnosis in patients with refractory lung abscesses.

PPL is defined as clonal lymphoid proliferation that affects one or both lungs (parenchyma and/or bronchi) in patients with no detectable extrapulmonary involvement at the time of diagnosis or during the subsequent three months (6). PPL is a rare tumor, comprising 0.45% of pulmonary malignant tumors, <1% of all lymphomas and 3.6% of extranodal lymphomas (1,6,7). Mucosa-associated lymphoid tissue lymphoma is the most frequent type of PPL, accounting for 58–87% of cases, whereas DLBCL reportedly accounts for 5–20% of cases (2,7,9,10).

As PPL is difficult to diagnose, the majority of diagnoses are made incidentally; 90% by surgical intervention and only 10% by non-surgical procedures, such as transbronchial lung biopsy or CT-guided percutaneous needle biopsy (5). Two cases of PPL that were difficult to diagnose and were consequently treated as other diseases have previously been reported (11,12). In one case, a transbronchial lung biopsy and CT-guided percutaneous needle biopsy failed to yield a diagnosis, and treatment of the apparent lung abscess was ineffective. Surgery was therefore performed, resulting in a diagnosis of PPL (11). In the other case, a transbronchial biopsy suggested a diagnosis of granulomatosis with polyangiitis, and sulfamethoxazole-trimethoprim therapy was temporarily effective. However, subsequent to the appearance of new nodular lesions, a final diagnosis of PPL was established by open biopsy (12). In the present case, the poor general condition of the patient precluded surgery, making the diagnosis more difficult.

PPL has diverse radiological findings, and 70–79% of patients reportedly present with multiple lesions (13). PPL spreads without destroying the existing lung structure, air bronchograms often being found within these tumors (2). There is generally no evidence of tissue destruction and the formation of cavities is rare. However, as in this case, formation of cavities reportedly occurs most often in DLBCL and is associated with a poor prognosis (14). In the present patient, a large cavitated mass was found, with no air bronchogram and with destruction of the existing lung structure. Thus, the typical radiological findings of PPL were absent. By the third admission, the lung mass had infiltrated the blood vessels, resulting in the so-called CT-angiogram sign, which is diagnostic of PPL (15). As illustrated by this case, the differential diagnosis is extremely important, particularly in patients with apparent lung abscesses (11,14).

The diagnosis of PPL of DLBCL type was finally made on the third admission. As this type of PPL has a poor prognosis, combination chemotherapy regimens are often administered following surgical resection with curative intent (2). With regard to the present study, the general condition of the patient was so poor upon first presentation to the Kobe City Medical Center General Hospital that aggressive therapy would not have been administered even if the correct diagnosis had been made at that time. The patient was initially diagnosed with a lung abscess and later with a refractory lung abscess. Pyothorax-associated lymphoma also may not have been ruled out upon first presentation. However, the patient did not display a typical medical history, such as evidence of tuberculosis pleuritis or an artificial pneumothorax (16). Also, a tissue sample could not be obtained by biopsy and repeated examination of the cytology revealed no evidence of malignancy. As there was videofluorography evidence of repeated aspiration, it was understandable that worsening aspiration pneumonia with formation of a lung abscess was clinically diagnosed. However, this case is instructive, as an earlier diagnosis may have been made if PPL had been considered in the differential diagnosis.

In conclusion, it should be recognized that PPL can mimic a lung abscess and that this diagnosis should consequently be one of the differential diagnoses of a refractory lung abscess.
